# Local Rather than Global H3K27me3 Dynamics Are Associated with Differential Gene Expression in Verticillium dahliae

**DOI:** 10.1128/mbio.03566-21

**Published:** 2022-02-08

**Authors:** H. Martin Kramer, Michael F. Seidl, Bart P. H. J. Thomma, David E. Cook

**Affiliations:** a Laboratory of Phytopathology, Wageningen University and Research, Wageningen, The Netherlands; b Theoretical Biology and Bioinformatics Group, Department of Biology, Utrecht University, Utrecht, The Netherlands; c University of Cologne, Institute for Plant Sciences, Cluster of Excellence on Plant Sciences (CEPLAS), Cologne, Germany; d Department of Plant Pathology, Kansas State University, Manhattan, Kansas, USA; INRA; Universidad de Córdoba

**Keywords:** epigenetics, fungus, histone modification, transcription

## Abstract

Differential growth conditions typically trigger global transcriptional responses in filamentous fungi. Such fungal responses to environmental cues involve epigenetic regulation, including chemical histone modifications. It has been proposed that conditionally expressed genes, such as those that encode secondary metabolites but also effectors in pathogenic species, are often associated with a specific histone modification, lysine27 methylation of H3 (H3K27me3). However, thus far, no analyses on the global H3K27me3 profiles have been reported under differential growth conditions in order to assess if H3K27me3 dynamics govern differential transcription. Using chromatin immunoprecipitation sequencing (ChIP-seq) and RNA sequencing data from the plant-pathogenic fungus Verticillium dahliae grown in three *in vitro* cultivation media, we now show that a substantial number of the identified H3K27me3 domains globally display stable profiles among these growth conditions. However, we observe local quantitative differences in H3K27me3 ChIP-seq signals that are associated with a subset of differentially transcribed genes between media. Comparing the *in vitro* results to expression during plant infection suggests that *in planta*-induced genes may require chromatin remodeling to achieve expression. Overall, our results demonstrate that some loci display H3K27me3 dynamics associated with concomitant transcriptional variation, but many differentially expressed genes are associated with stable H3K27me3 domains. Thus, we conclude that while H3K27me3 is required for transcriptional repression, it does not appear that transcriptional activation requires the global erasure of H3K27me3. We propose that the H3K27me3 domains that do not undergo dynamic methylation may contribute to transcription through other mechanisms or may serve additional genomic regulatory functions.

## INTRODUCTION

The fungal kingdom comprises a plethora of species occupying an enormously diverse range of ecological niches (1). As environments are typically dynamic, including the effects of daily and yearly cycles, fungi continuously need to respond and adapt to survive (2, 3). To this end, fungi have evolved various mechanisms to monitor their environment and transcriptionally respond to environmental cues (4). For instance, the yeast Saccharomyces cerevisiae senses cold stress by increased membrane rigidity, which leads to the transcription of genes that, among others, encode cell damage-preventing proteins (5). Furthermore, S. cerevisiae senses the quantitative availability of carbon and nitrogen sources in the environment to determine which developmental program maximizes the potential for survival (6). In animal systems, epigenetic mechanisms (i.e., those affecting the genetic output without changing the genetic sequence) are implicated in transcriptional responses to changing environments (7–9). Such epigenetic mechanisms have similarly been proposed to contribute to environmental responses in filamentous fungi. For instance, the saprotrophic fungus Neurospora crassa phenotypically reacts to environmental stimuli such as changes in temperature and pH, yet N. crassa mutants impaired in transcription-associated epigenetic mechanisms display reduced growth in response to these stimuli (10). Similarly, the nectar-feeding yeast Metschnikowia reukaufii and the ubiquitous fungus Aureobasidium pullulans fail to properly respond to changing carbon sources when DNA methylation or histone acetylation is inhibited (11, 12). These results suggest that epigenetic mechanisms are important for transcriptional responses to changing environments in diverse fungi, but many questions remain regarding the precise mechanisms and function of epigenetic dynamics in fungi.

Epigenetic mechanisms, such as direct modifications of DNA and histone proteins or physical changes to the chromatin architecture, can influence transcription by regulating DNA accessibility (13). Chromatin that is accessible and potentially active is termed euchromatin, while heterochromatin is condensed and often transcriptionally silent (14). However, heterochromatic regions are not always repressed. Heterochromatin is subcategorized into constitutive heterochromatin that remains condensed throughout the cell cycle and facultative heterochromatin that can decondense to allow transcription in response to developmental changes or environmental stimuli (15–17). In fungi, constitutive heterochromatin is often associated with repeat-rich genome regions and is typically characterized by the trimethylation of lysine 9 on histone H3 (H3K9me3), while facultative heterochromatin is characterized by the trimethylation of lysine 27 on histone H3 (H3K27me3) (18–21). Empirically, both H3K9me3 and H3K27me3 have been implicated in transcriptional regulation in various fungi (22–32). The majority of these studies rely on genetic perturbation of the enzymes that deposit methylation at H3K9 and H3K27, and the results consistently show that the depletion of methylation at these lysine residues mainly results in transcriptional induction. However, as the global depletion of a histone modification can result in pleiotropic effects, such as the improper localization of other histone modifications or altered development (33, 34), it is difficult to infer transcriptional control mechanisms used for natural gene regulation from these genetic perturbation experiments. Therefore, additional research is needed to directly test the hypothesis that heterochromatin-associated histone modifications directly regulate transcription through either their dynamics or their action to form or recruit transcriptional complexes (35–37).

The filamentous fungus Verticillium dahliae is a soilborne broad-host-range plant pathogen that infects plants through the roots to invade the xylem vessels and cause vascular wilt disease (38). Genomic and transcriptomic studies have revealed that the V. dahliae genome harbors lineage-specific (LS) regions that are variable between strains and enriched for genes that are *in planta* induced (39, 40). These LS regions are generally considered genomic hot spots for evolutionary adaptation to plant hosts (39–44). Recently, we explored the epigenome of V. dahliae and distinguished LS regions from the core genome based on particular chromatin signatures, including elevated levels of H3K27me3 accompanied by accessible DNA and active transcription (44). Using a machine learning approach and supported by orthogonal analyses, we identified nearly twice as much LS DNA as previously considered, collectively referred to as adaptive genomic regions (44). Given the elevated levels of H3K27me3 at adaptive genomic regions in V. dahliae and previous reports that the removal of H3K27me3 results in transcriptional induction (22–24), we now tested if H3K27me3 dynamics are required for the transcriptional activation of genes under different growth conditions. Ideally, the involvement of H3K27me3 dynamics in the differential gene expression of V. dahliae would be studied between *in vitro* and *in planta* growth, as adaptive genomic regions are enriched for *in planta*-induced genes (40, 44). However, V. dahliae displays a low pathogen-to-plant biomass during infection (45), which impedes technical procedures to determine histone modification levels over the genome (46). Nevertheless, as H3K27me3 is generally reported to regulate transcription in response to environmental stimuli (15, 17), we hypothesize that it may be involved in transcriptional regulation in V. dahliae under differential growth conditions *ex planta* as well. Here, we analyze RNA sequencing (RNA-seq), chromatin immunoprecipitation sequencing (ChIP-seq), and assay for transposase accessible chromatin followed by sequencing (ATAC-seq) data for V. dahliae cultured in various axenic growth media to understand if transcriptional dynamics require concomitant changes in the H3K27me3 modification status.

## RESULTS

### Chromatin features correlate with gene expression levels.

**FIG 1:**
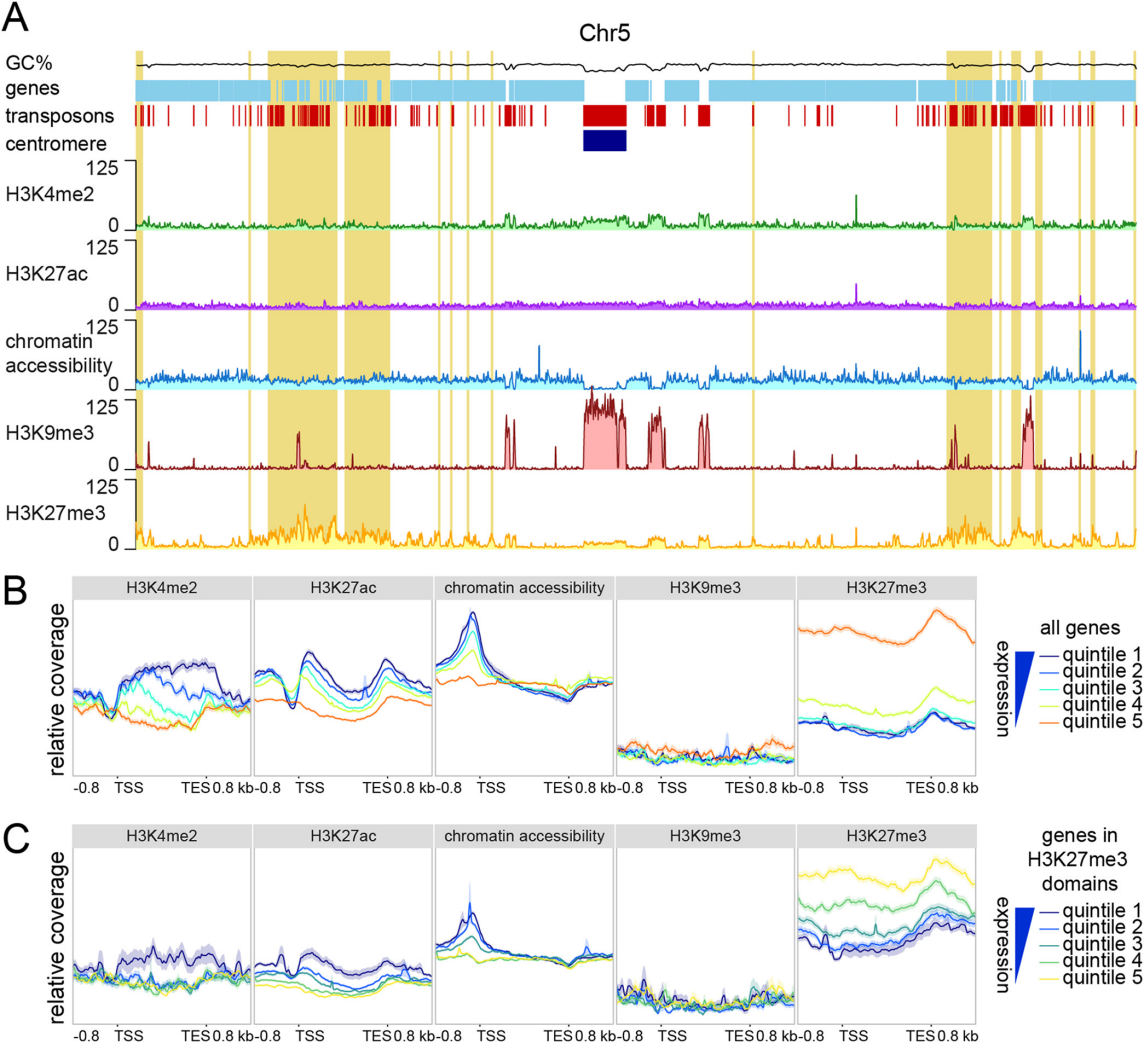
Lowly expressed and nonexpressed genes associated with H3K27me3. (A) Whole-genome distribution of the euchromatin-associated histone modifications H3K4me2 and H3K27ac, the constitutive heterochromatin-associated histone modification H3K9me3, the facultative heterochromatin-associated histone modification H3K27me3, and chromatin accessibility as determined by ATAC-seq, based on chromosome 5 as an example. The percent GC content is indicated in black, genes are indicated in light blue, transposons are indicated in red, the centromere is indicated in dark blue, and adaptive genomic regions are indicated in yellow. (B and C) Relative coverage of chromatin accessibility and the histone marks H3K4me2, H3K9me3, and H3K27me3 over gene bodies (between the transcription start site [TSS] and the transcription end site [TES]), ±800 bp of flanking sequence, grouped into quintiles based on gene expression levels upon cultivation for 6 days in potato dextrose broth (PDB) for all genes (B) and for all genes located in an H3K27me3 domain (C).

To determine how general features of chromatin, such as histone modifications and DNA accessibility, impact transcriptional activity in V. dahliae, we mapped the occurrence of the heterochromatic histone marks H3K9me3 and H3K27me3, the euchromatic histone marks dimethylation of lysine 4 on histone H3 (H3K4me2) and acetylation of lysine 27 on histone H3 (H3K27ac), and chromatin accessibility determined by ATAC-seq (47) (Fig. 1A; see also Fig. S1 in the supplemental material). Grouping the V. dahliae genes into five expression quintiles, from quintile 1, containing the 20% most highly expressed genes, to quintile 5, with the 20% most lowly expressed genes, we are able to integrate histone modification profiles and transcriptional activity. Expressed genes (quintiles 1 to 4) displayed low H3K4me2 and H3K27ac coverage upstream of the transcription start site (TSS), followed by a steep increase of coverage over the start of the gene that decreased over the gene body and increased again at the transcription end site (TES) (Fig. 1B). The strength of the association corresponds to the level of transcription, also within quintiles (Fig. S2). The low H3K4me2 and H3K27ac coverage directly upstream of the TSS coincides with increased chromatin accessibility, where more highly expressed quintiles have more open DNA (Fig. 1B; Fig. S2). This chromatin profile upstream of the TSS suggests the occurrence of a nucleosome-depleted region. There is little evidence of H3K9me3 over gene bodies and *cis*-regulatory regions (Fig. 1B; Fig. S2), which corroborates that H3K9me3 marks TESs in constitutive heterochromatic regions such as the centromeres (18, 44, 48, 49). During cultivation in potato dextrose broth (PDB), H3K27me3 is mainly present on genes that are lowly or not expressed (quintiles 4 and 5) (Fig. 1B; Fig. S2). These results, and the association between chromatin features and transcriptional activity, are consistent with previous reports for other fungi (24, 27, 32).

10.1128/mbio.03566-21.1 FIG S1 Distribution of chromatin features over all chromosomes. Download FIG S1, PDF file, 0.3 MB. Copyright © 2022 Kramer et al. 2022 Kramer et al. https://creativecommons.org/licenses/by/4.0/ This content is distributed under the terms of the Creative Commons Attribution 4.0 International license.

10.1128/mbio.03566-21.2 FIG S2 Gene expression correlates with histone modification presence and chromatin accessibility. Download FIG S2, PDF file, 0.2 MB. Copyright © 2022 Kramer et al. 2022 Kramer et al. https://creativecommons.org/licenses/by/4.0/ This content is distributed under the terms of the Creative Commons Attribution 4.0 International license.

To further analyze the association between H3K27me3 and gene expression, we identified 3,186 genes covered by H3K27me3 peaks (see Materials and Methods) from triplicate-grown V. dahliae in PDB. These 3,186 genes were separated into five expression quintiles as described above. Interestingly, we found that genes in H3K27me3 domains with higher expression values (quintiles 1 and 2) had higher H3K4me2 values over the gene body, more accessible promoters, and lower H3K27me3 values (Fig. 1C). This suggests that genes in H3K27me3 domains are not uniformly repressed or heterochromatic, and there appears to be a quantitative, rather than a qualitative, association between H3K27me3 association and gene activity. Genes with a lower association with H3K27me3 may represent loci that are not in a stable state under the tested conditions, where some cells have a more euchromatic profile and others have a more heterochromatic profile. While we cannot infer these details from the current data, it is clear that genes with lower expression levels in PDB are generally marked with H3K27me3, have less H3K4me3, and have less accessible DNA in the region of transcription initiation (i.e., their promoter).

### Genetic perturbation of H3K27me3 induces the transcription of many genes that are differentially expressed *in vitro* and *in planta*.

**FIG 2:**
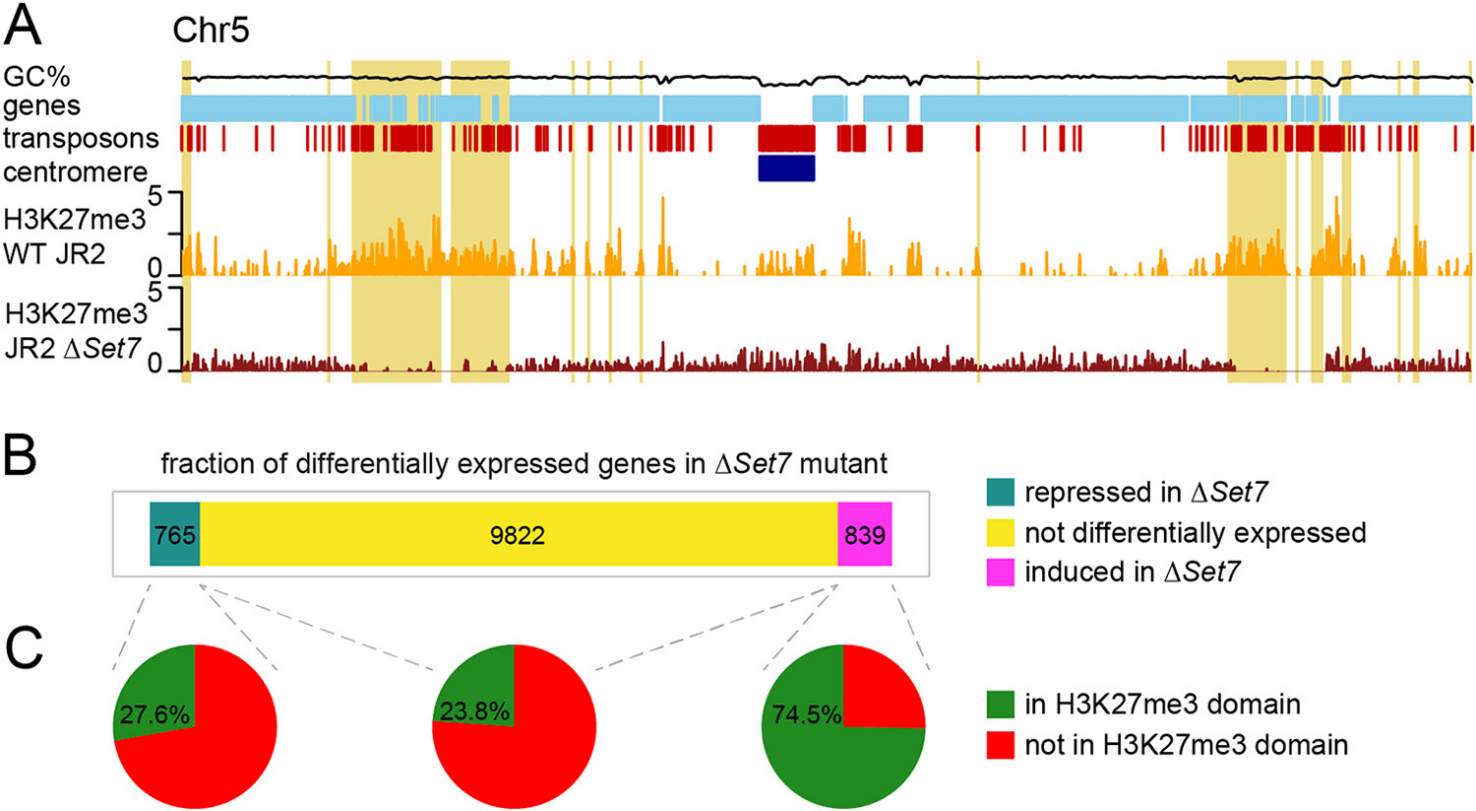
Genetic perturbation of H3K27me3 results in the induction of genes that are transcriptionally regulated under different growth conditions. (A) H3K27me3 ChIP sequencing on triplicate WT JR2 (yellow) and duplicate Δ*Set7* (red) samples with coverage over chromosome 5 as an example. Adaptive genomic regions are indicated in yellow. (B and C) Fractions of induced (log_2_ fold change of greater than 1 [*P* < 0.05]) and repressed (log_2_ fold change of less than −1 [*P* < 0.05]) genes and genes that are not differentially expressed between the wild-type and Δ*Set7* strains (B) and those that are located in H3K27me3 domains (C).

To further characterize the influence of H3K27me3 on gene expression in V. dahliae, we deleted the histone methyltransferase component of polycomb repressive complex 2 (PRC2), termed *Set7* (Δ*Set7*), leading to the loss of H3K27me3 (Fig. 2A; Fig. S3 and S4). We note that some background signal is present for the H3K27me3 ChIP-seq conducted in the Δ*Set7* mutant, but the signal is relatively uniform across the genome and does not correspond to the regions of H3K27me3 found in the wild type (WT) (Fig. 2A). As H3K27me3 is generally associated with facultative heterochromatin, we anticipated that the loss of H3K27me3 would mainly lead to the induction of genes that were located in H3K27me3 domains in wild-type V. dahliae. Out of the 839 genes that are induced in the Δ*Set7* strain (log_2_ fold change of >2; *P* < 0.05), 625 (74.5%) are located in H3K27me3 domains, which is significantly higher than expected given that only 27.9% of genes are located in H3K27me3 domains (*P* < 0.00001 by Fisher’s exact test). In contrast, we find that 211 (27.6%) of 765 repressed genes in the Δ*Set7* mutant are in H3K27me3 domains (no association; *P* = 0.94 by Fisher’s exact test) (Fig. 2B and C). Additionally, when comparing log_2_ fold changes in expression between the Δ*Set7* and wild-type strains, we observed that genes located in an H3K27me3 domain in the wild type are more significantly induced in the Δ*Set7* mutant than genes not located in H3K27me3 domains (*P* < 0.001 by two-sample Student’s *t* test) (Fig. S5). These findings support the role of H3K27me3 in transcriptional repression and show that the loss of H3K27me3 can lead to derepression during growth *in vitro*.

10.1128/mbio.03566-21.3 FIG S3 Western blot showing the loss of H3K27me3 in the V. dahliae Δ*Set7* deletion mutant. Download FIG S3, PDF file, 0.06 MB. Copyright © 2022 Kramer et al. 2022 Kramer et al. https://creativecommons.org/licenses/by/4.0/ This content is distributed under the terms of the Creative Commons Attribution 4.0 International license.

10.1128/mbio.03566-21.4 FIG S4 ChIP sequencing shows the loss of H3K27me3 in the V. dahliae Δ*Set7* mutant. Download FIG S4, PDF file, 0.1 MB. Copyright © 2022 Kramer et al. 2022 Kramer et al. https://creativecommons.org/licenses/by/4.0/ This content is distributed under the terms of the Creative Commons Attribution 4.0 International license.

10.1128/mbio.03566-21.5 FIG S5 Genes associated with H3K27me3 in wild-type V. dahliae are more strongly transcriptionally induced in the Δ*Set7* mutant than non-H3K27me3-associated genes. Download FIG S5, PDF file, 0.06 MB. Copyright © 2022 Kramer et al. 2022 Kramer et al. https://creativecommons.org/licenses/by/4.0/ This content is distributed under the terms of the Creative Commons Attribution 4.0 International license.

### H3K27me3 domains are globally stable between *in vitro* growth conditions.

**FIG 3:**
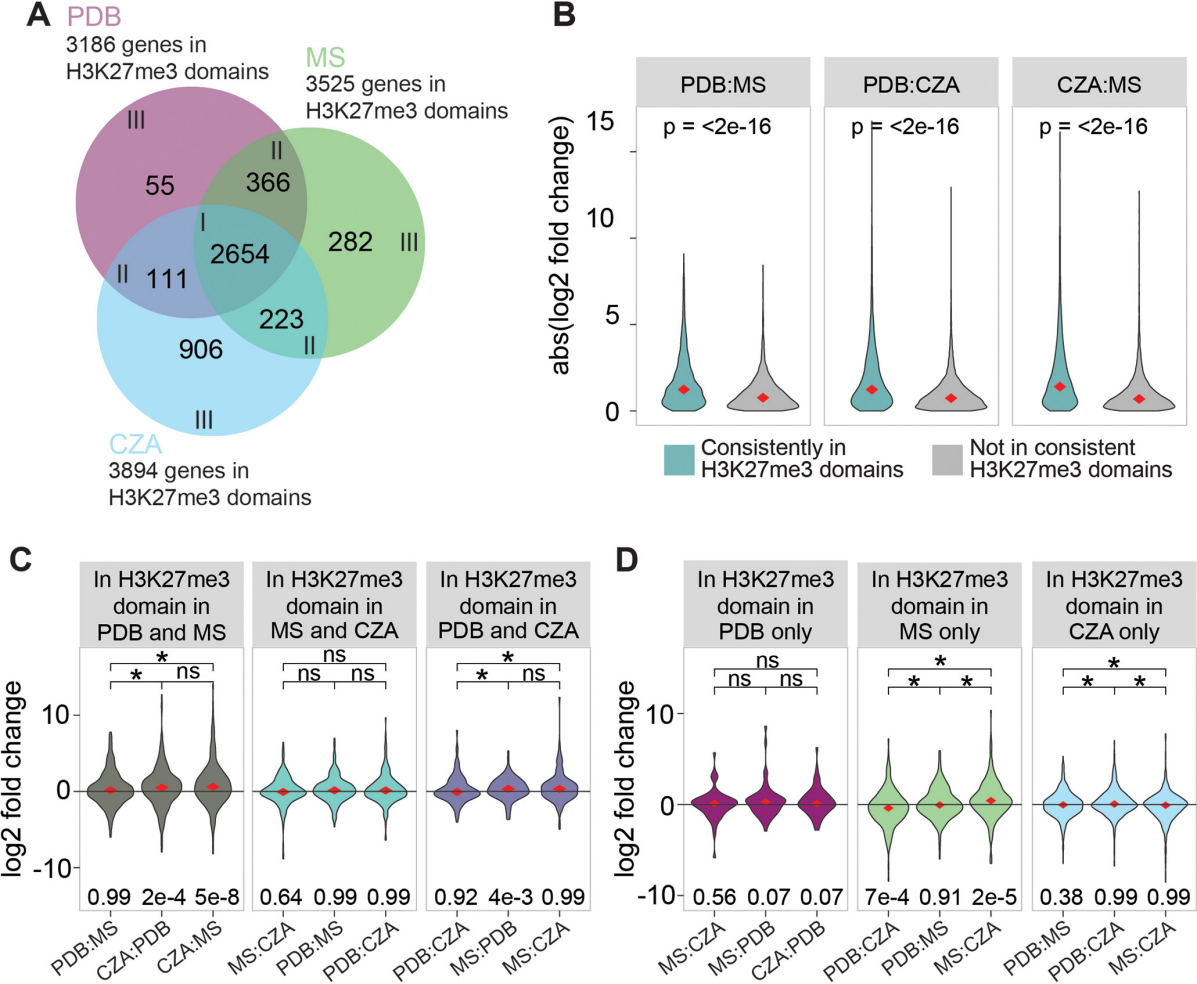
Differential H3K27 methylation partially explains differential expression. (A) Numbers of genes located in H3K27me3 domains for V. dahliae cultivated for 6 days in Czapek-Dox medium (CZA), half-strength Murashige-Skoog medium (MS), and potato dextrose broth (PDB). (B) Absolute (abs) log_2_ fold changes in expression between pairs of growth media. The genes were grouped between those consistently localized in H3K27me3 domains (2,654 genes) and those not consistently present in H3K27me3 domains. (C and D) Log_2_ fold change values for each pair of growth media for genes in H3K27me3 domains found in two (C) or one (D) of three tested *in vitro* growth media. Differential gene expression comparisons are set such that genes more highly expressed in the medium in which they are not located in the H3K27me3 domain will have a positive log_2_ fold change. In cases where differential gene expression comparisons are between growth media in which the genes are located in H3K27me3 domains in both (C) or neither (D) of the compared media, the orientation of positive and negative log_2_ fold changes is arbitrary. Median values of violin plots are indicated with diamonds and shown above the plot. Significant differences in TPM values between growth media were determined by the one-sample Wilcoxon signed-rank test (*, *P* ≤ 0.05; ns, not significant). A one-sample two-sided *t* test was used to test whether sample means significantly differ from zero. *P* values are shown below the plot.

**FIG 4:**
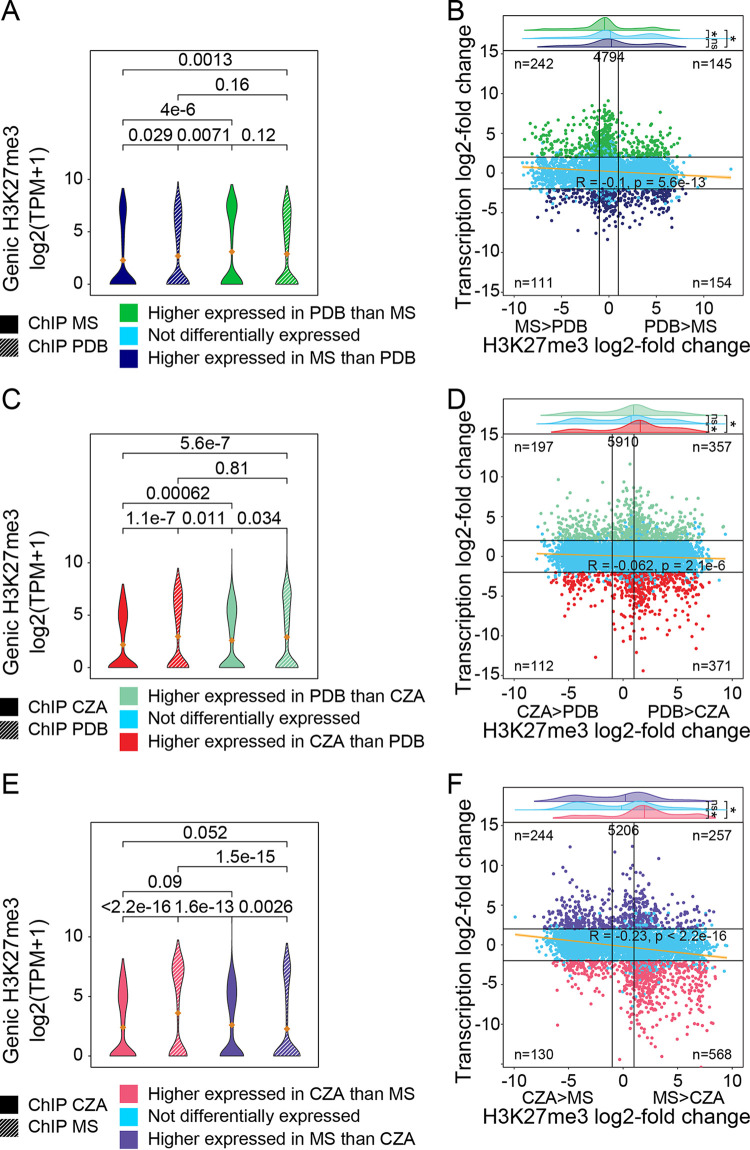
Differentially expressed genes associated with local changes in H3K27me3 coverage. Shown are pairwise comparisons of input-corrected H3K27me3 coverage over differentially expressed genes for V. dahliae cultivated for 6 days in half-strength Murashige-Skoog medium (MS) and potato dextrose broth (PDB) (A and B), Czapek-Dox medium (CZA) and PDB (C and D), and MS and CZA (E and F). (A, C, and E) H3K27me3 coverage over differentially expressed genes in each corresponding growth medium. Mean values of violin plots are indicated with orange diamonds. (B, D, and F) Per-gene comparison of log_2_ fold changes in transcription and genic H3K27me3 coverage for genes with input-corrected H3K27me3 coverage of >0 in either or both of the compared media (the total number of genes is indicated at the top of the plot). Black horizontal lines indicate transcription log_2_ fold changes of −2 and 2, and black vertical lines indicate H3K27me3 log_2_ fold changes of −1 and 1. The numbers of genes in the four-corner section are indicated. Significant differences in TPM values between growth media or gene sets were determined by the one-sample Wilcoxon signed-rank test and are indicated by their *P* value or by asterisks (*, *P* ≤ 0.05).

Given that H3K27me3 domains contribute to transcriptional repression, a key question concerns the status of H3K27me3 under growth conditions where the underlying genes are transcribed. One hypothesis is that H3K27me3 is removed or lost under growth conditions that activate gene expression, which would be noticeable as a change in H3K27me3 ChIP-seq domains between V. dahliae growth conditions that lead to differential expression. Here, the observed changes in H3K27me3 domains should be associated with transcriptional differences of the underlying genes. An alternative hypothesis is that the H3K27me3 domain status does not change in accordance with transcriptional activity, and the repressive effects of H3K27me3 are released through alternative means. To test this hypothesis, we performed triplicates of H3K27me3 ChIP-seq on V. dahliae cultivated for 6 days in Murashige-Skoog medium (MS) and Czapek-Dox medium (CZA), in addition to the previously generated ChIP-seq data in PDB. Based on the correlation between replicates (Fig. S6), we decided to continue with 3 replicates of H3K27me3 ChIP data in PDB and 2 replicates each of H3K27me3 ChIP data in CZA and MS. Control ChIP input samples were used to normalize H3K27me3 data sets and identify H3K27me3 domains in each of the three growth media. We identified a total of 2,654 genes that were always present in H3K27me3 domains, regardless of the *in vitro* growth media (Fig. 3A). Interestingly, the 2,654 genes present in stable H3K27me3 domains display significantly stronger differential expression between all pairwise medium comparisons than nonmarked genes under all three growth conditions (Fig. 3B). This suggests that differential gene expression can occur without changes in global H3K27me3 coverage. We further checked whether genes that are differentially expressed between *in vitro* growth media are associated with H3K27me3 during cultivation in the growth medium with low expression levels (Fig. S7). Interestingly, we observed that for all pairwise comparisons between growth media, genes with higher log_2_ fold changes in expression are more likely to be located in an H3K27me3 domain in the nontranscriptionally permissive growth medium (Fig. S7), again suggesting that H3K27me3 is involved in the regulation of differential gene expression. To assess if changes in H3K27me3 domains between the media were associated with changes in transcription, we compared differences in domains and differential gene expression between media. We first analyzed differential expression for genes present in H3K27me3 domains in two media but not in an H3K27me3 domain in the third medium. For example, we identified 366 genes that were in an H3K27me3 domain in PDB and MS but not during CZA growth (Fig. 3A) and found that these genes are not differentially expressed between PDB and MS (*P* = 0.99 by a one-sample *t* test), but they are more highly expressed in CZA than in both PDB (*P* = 2e−4 by a one-sample *t* test) and MS (*P* = 5e−8 by a one-sample *t* test) (Fig. 3C). Similarly, genes present in shared H3K27me3 domains for CZA and PDB growth (111 genes) are not differentially expressed between CZA and PDB (*P* = 0.92 by a one-sample *t* test); when the genes are associated with H3K27me3, they are more highly expressed in MS than in PDB (*P* = 4e−3 by a one-sample *t* test) but not more highly expressed in MS than in CZA (*P* = 0.99 by a one-sample *t* test). For genes present in shared H3K27me3 domains between MS and CZA growth (223 genes), we did not observe statistically significant transcriptional differences between the growth conditions where the genes lacked H3K27me3 domains (Fig. 4A and C). Analyzing H3K27me3 domains unique to a medium, we found that CZA growth had the highest number and proportion of unique genes located in H3K27me3 domains (23.3%), followed by 8.0% unique to MS growth and 1.7% unique to PDB growth (Fig. 3A). The genes uniquely marked under any condition did not show consistently increased expression under the condition in which the gene was not located in an H3K27me3 domain (Fig. 3D). Overall, these results suggest that differential expression can be associated with differential H3K27me3 domain status, but it is not a requirement. We observed clear examples where the loss of H3K27me3 in one medium is associated with increased transcription in that medium, but this was not universally true. Many genes undergo differential gene expression between growth conditions and remain in stable H3K27me3 domains. We note that the majority of genes located in H3K27me3 domains were common to all three growth conditions, accounting for 83.3%, 75.3%, and 68.2% of the identified genes in H3K27me3 domains from PDB, MS, and CZA growth, respectively, indicating that the qualitative presence of H3K27me3 domains is globally stable.

10.1128/mbio.03566-21.6 FIG S6 Correlation between H3K27me3 ChIP samples. Download FIG S6, PDF file, 0.10 MB. Copyright © 2022 Kramer et al. 2022 Kramer et al. https://creativecommons.org/licenses/by/4.0/ This content is distributed under the terms of the Creative Commons Attribution 4.0 International license.

10.1128/mbio.03566-21.7 FIG S7 Differentially expressed genes are enriched in H3K27me3 domains during cultivation in nonpermissive growth medium. Download FIG S7, PDF file, 0.1 MB. Copyright © 2022 Kramer et al. 2022 Kramer et al. https://creativecommons.org/licenses/by/4.0/ This content is distributed under the terms of the Creative Commons Attribution 4.0 International license.

### Local quantitative differences in H3K27me3 levels are associated with transcriptional differences.

Our analysis of H3K27me3 presence/absence dynamics did not account for potential quantitative differences between growth media. Whole-chromosome plots of H3K27me3 domains identified between media reflect their generally stable presence (Fig. S8), but the analysis is based on qualitative H3K27me3 domain identification. Domain calling for H3K27me3 results in broad domains, but this fails to capture higher-resolution quantitative differences that may exist between media, as is seen during an inspection of global chromosome plots (Fig. S8). To understand how qualitative domain calling impacts the analysis, we examined quantitative differences between H3K27me3 ChIP-seq signals and transcriptional outputs between pairs of growth conditions. Genes were grouped based on differential gene expression between media, where genes that were significantly more highly expressed in medium A were one group and genes that were significantly more highly expressed in medium B were another group. Subsequently, the H3K27me3 ChIP-seq signals relative to the input samples were normalized and compared between both growth media for the groups of differentially expressed genes (Fig. 4). Comparing the results for PDB versus MS, we see that genes that are more highly expressed in MS have a significantly lower MS H3K27me3 ChIP signal than the same genes from PDB ChIP (Fig. 4A). The contrasting comparison, genes that are more highly expressed in PDB, shows that these genes do not significantly differ in ChIP signals between PDB and MS growth (Fig. 4A). Further integrating the transcriptional and ChIP fold changes, for the 4,794 genes that have an input-corrected H3K27me3 ChIP signal above zero in either MS or PDB, we see that genes more highly expressed in PDB have a significantly lower log_2_ fold change for H3K27me3 coverage, indicating that these genes have lower H3K27me3 signals in PDB than in MS (Fig. 4B). Quantifying the numbers of genes in quadrants II and IV, we find that 396 (242 + 154) display a negative association between transcription and H3K27me3 ChIP signals, whereas 256 (145 + 111) display a positive association (Fig. 4B). Thus, more genes are present in the quadrants that represent genes having lower H3K27me3 levels and higher transcription levels between the two growth conditions. The linear regression based on all genes is an *R* value of −0.1, also suggesting the low but significant negative association (Fig. 4B). It is clear that the association between differential expression and changes in ChIP signals is not true for all genes, but overall, we observe that the majority of genes that display changes for H3K27me3 ChIP signals between media show the predicted transcriptional response where less H3K27me3 is associated with higher transcription levels (Fig. 4B).

10.1128/mbio.03566-21.8 FIG S8 Distribution of H3K27me3 for V. dahliae cultivated in *in vitro* growth media. Download FIG S8, PDF file, 0.2 MB. Copyright © 2022 Kramer et al. 2022 Kramer et al. https://creativecommons.org/licenses/by/4.0/ This content is distributed under the terms of the Creative Commons Attribution 4.0 International license.

The results for PDB versus CZA growth showed that genes more highly expressed in CZA have higher H3K27me3 levels in PDB, consistent with the expected association (Fig. 4C). For genes more highly expressed in PDB, however, we also observed a higher H3K27me3 level in PDB. Globally, the data indicated that genes more highly expressed in CZA have more H3K27me3 ChIP signals in PDB than genes that are more highly expressed in PDB, indicating a negative association between transcription and H3K27me3 presence (Fig. 4D). This is corroborated by the number of genes per quadrant, as 568 (197 + 371) genes are located in quadrants II and IV, whereas 469 (112 + 357) genes are located in quadrants I and III (Fig. 4D). The linear regression analysis indicates a slight negative association between differential expression and the H3K27me3 ChIP signal (Fig. 4D). We also observed an overall higher ChIP signal from samples grown in CZA, but the reason for this is not clear.

The results for CZA versus MS growth showed that genes more highly expressed in CZA had statistically higher levels of H3K27me3 ChIP signals in MS, whereas genes more highly expressed in MS had higher levels of H3K27me3 in CZA (Fig. 4E). The same pattern was observed in the integrated analysis, as genes more highly expressed in CZA had more H3K27me3 signal in MS (Fig. 4F). There are 812 (244 + 568) genes in quadrants II and IV, supporting a negative association between differential transcription and the H3K27me3 ChIP signal, whereas 387 (130 + 257) genes are present in quadrants I and III. This is supported by the significant negative correlation (*R* = −0.23) (Fig. 4F). Overall, the results of the integrated analyses show that there is an association between quantitative transcriptional levels and H3K27me3 signals, where genes that are more highly expressed in a transcriptionally permissive medium have lower H3K27me3 than H3K27me3 levels in the repressive media. There are also many genes that were differentially expressed without an accompanying shift in the H3K27me3 log_2_ fold change (Fig. 4B, D, and F). Collectively, these results are consistent with the observations of the qualitative H3K27me3 presence/absence comparisons and suggest that H3K27me3 levels are generally stable, but local changes at particular genes may contribute to transcriptional dynamics.

### Genes induced *in planta* are largely H3K27me3 associated across all tested growth conditions.

**FIG 5:**
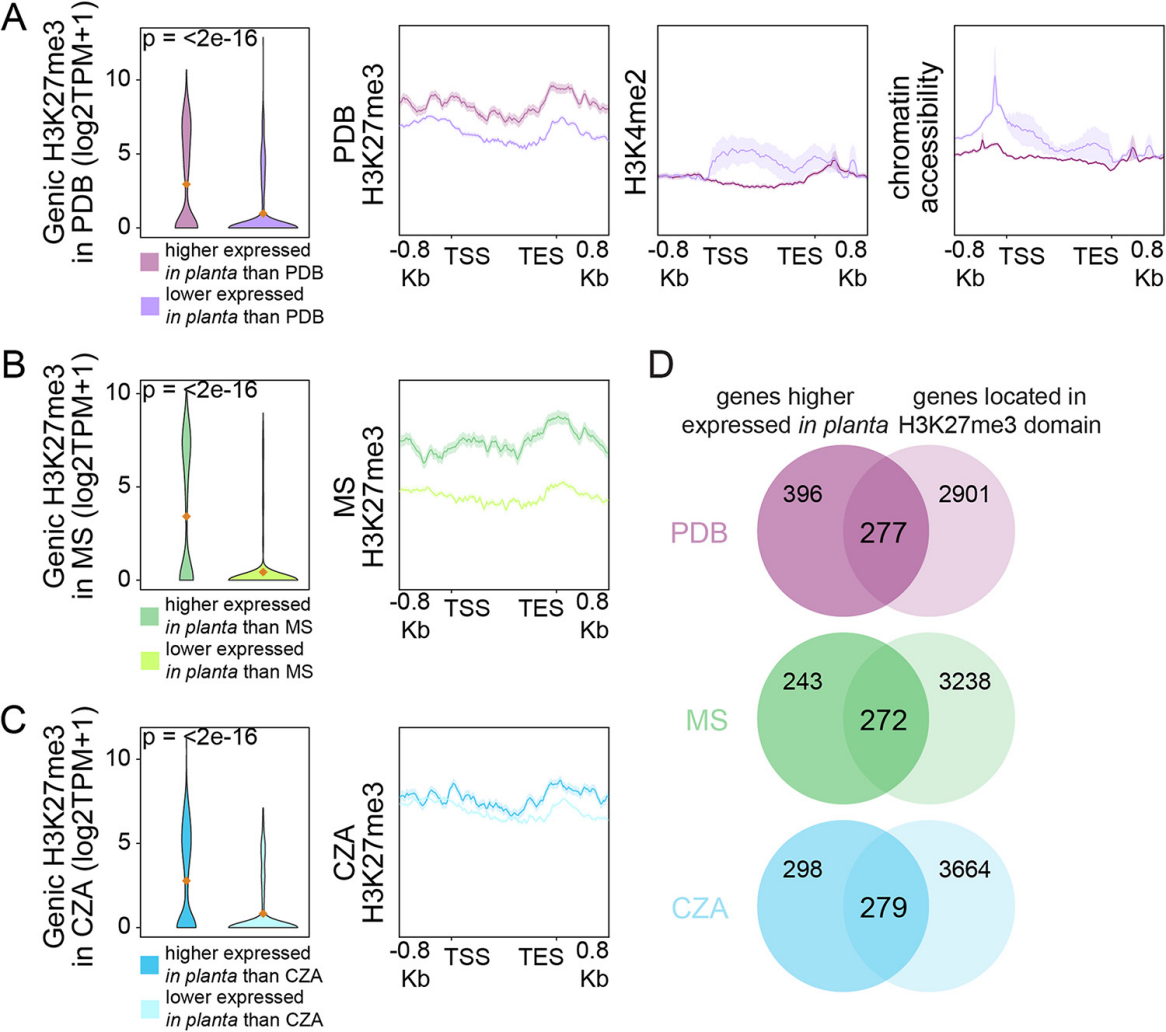
*In planta*-induced genes are H3K27me3 associated under transcriptionally repressive conditions. (A to C) Violin plots displaying input-corrected H3K27me3 ChIP signals over genes differentially expressed between *in planta* and 6 days of cultivation in potato dextrose broth (PDB) (A), half-strength Murashige-Skoog medium (MS) (B), and Czapek-Dox medium (CZA) (C). Mean values are indicated by orange diamonds. Line plots display average coverages of chromatin features over the gene sets. (D) Overlap of genes that are more highly expressed *in planta* than in PDB, MS, or CZA with genes located in an H3K27me3 domain in the corresponding growth medium. Significant differences in H3K27me3 coverage were determined by the one-sample Wilcoxon signed-rank test (*, *P* ≤ 0.05).

The presented analyses compared the directionality of H3K27me3 and transcriptional changes between axenic growth media to address if H3K27me3 dynamics are associated with transcriptional dynamics. Another interesting growth condition for V. dahliae is host colonization and the contrast for the genes differentially expressed *in planta* compared with axenic culture. We have thus far been unable to perform ChIP on V. dahliae during host colonization due to a low pathogen-to-plant biomass, and therefore, we cannot compare H3K27me3 levels between these conditions. However, we can identify the genes that are significantly induced during infection compared to *in vitro* growth media and assess their chromatin profiles in these media to assess if *in planta*-induced genes appear heterochromatic during *in vitro* growth. Genes were grouped based on differential expression between PDB and *in planta* growth, and chromatin profiles showed that genes that are more highly expressed *in planta* have significantly more H3K27me3 during PDB growth than genes that are more highly expressed in PDB (Fig. 5A). These *in planta*-induced genes lacked H3K4me2, and the DNA was less accessible during PDB growth (Fig. 5A). The association between *in planta* induction and higher H3K27me3 levels was seen relative to not only PDB growth but also growth in MS and CZA (Fig. 5B and C). For each medium comparison, the genes that are more highly expressed *in planta* have higher H3K27me3 levels during axenic growth. To understand what genes are driving these differences, we investigated whether *in planta-*induced genes that are located in H3K27me3 domains are overrepresented for genes that are potentially involved in infection. There were approximately 600 genes that are more highly expressed *in planta* than in any of the three media (Fig. 5D). We found that, depending on the medium, from 41.2% to 52.8% of the *in planta*-induced genes were located in H3K27me3 domains (Fig. 5D). We observed that the *in planta-*induced genes within H3K27me3 domains have a higher, yet not statistically significant, fraction of genes encoding secreted proteins or putative effectors than all *in planta-*induced genes. For example, of the genes in H3K27me3 domains that were *in planta* induced compared with PDB growth (277 genes total), 15.5% had a secretion signal, and 4.3% were predicted effectors. This is compared to all *in planta*-induced genes relative to PDB (673 genes total), where 12.3% had a secretion signal and 2.7% were predicted effectors. These results were similar between the other two media, and we conclude that *in planta*-induced genes in H3K27me3 domains have a slightly higher fraction of genes that are potentially involved in infection than *in planta*-induced genes not in H3K27me3 domains. Collectively, these results indicate that genes that are more highly expressed *in planta* have a heterochromatic profile (i.e., H3K27me3 association, low H3K4me2, and lower accessibility) when analyzed in axenic culture. The chromatin profile of these genes during *in planta* growth will need to be directly assessed in the future.

## DISCUSSION

Chemical histone modifications play an essential role in transcriptional regulation, but a number of mechanistic questions remain for their role in differential transcription in filamentous fungi. In this study, we show that many genes that are differentially transcribed between *in vitro* growth conditions are located in H3K27me3 domains. This is interesting because the mark contributes to transcriptional repression, and it is relatively sparse in the genome, occupying approximately 33% of the genes. However, 35% to 70% of differentially expressed genes identified between growth conditions reside within H3K27me3 domains. It is not clear what mechanism might drive this association.

The global fraction of genes that are located in H3K27me3 domains in V. dahliae is similar to what has been reported for the ascomycete Podospora anserina (28) but higher than those for N. crassa, Zymoseptoria tritici, Leptosphaeria maculans, and Magnaporthe oryzae (9 to 16% of genes in H3K27me3 domains) (20, 30, 32, 50). Previous reports showed that H3K27me3 represses the transcription of secondary metabolite clusters in Fusarium spp. and Epichloë festucae and of effectors in Z. tritici and M. oryzae (22, 24, 31, 32, 50). However, these reports have mainly relied on genomic associations with H3K27me3, genetic perturbations altering H3K27me3 deposition, and analyses of only a few genes under natural conditions. Given our results and these previous findings, our hypothesis was that differences in transcription between *in vitro* growth conditions are in part coordinated by dynamics for H3K27 methylation. We directly addressed this phenomenon using genome-wide H3K27me3 profiling and RNA-seq under different *in vitro* growth conditions. Consistent with reports for other fungi, we see that H3K27me3 is associated with transcriptional repression and that deleting *Set7*, encoding the histone methyltransferase that is responsible for H3K27me3, leads to the induction of many genes normally present in H3K27me3 domains. Importantly, our direct observations of H3K27me3 levels and transcriptional output from three *in vitro* media showed that H3K27me3 domains are generally stable globally. We see that 50% to 75% of the identified H3K27me3 domains, depending on the medium analyzed, did not change between the tested conditions. Despite many H3K27me3 domains not undergoing presence/absence dynamics, numerous genes in these domains displayed differential transcription between the tested conditions, indicating that a complete loss of H3K27me3 is not strictly required for transcriptional induction.

One possibility to account for these seemingly contradictory results is that even though H3K27me3 appears stable at the level of broad-peak calling, there may be smaller regions within the broad domain that are dynamic. Assessing quantitative differences in H3K27me3 ChIP-seq levels at defined genomic locations between paired *in vitro* growth conditions indicates that this can account for some of the observed transcriptional differences. For example, some genes have a lower H3K27me3 ChIP signal upon growth in transcriptionally permissive medium than upon growth in transcriptionally repressive medium. We interpret these results as evidence for local, rather than global, changes in H3K27me3 dynamics contributing to transcriptional regulation for the underlying genes. H3K27me3-associated genes that are differentially expressed while the presence of the histone mark remains stable may be transcriptionally regulated through the activity of H3K27me3 readers. For instance, the Fusarium graminearum histone reader BP1, which is orthologous to N. crassa EPR-1, specifically binds to H3K27me3 and corepresses gene transcription (51, 52). The gene encoding BP1 is conserved within fungi, including V. dahliae (51). The dynamic binding of such a transcription-repressing histone reader to stable H3K27me3 domains may explain the observed transcriptional dynamics.

We note that an individual histone does not permit a quantitative trimethylation status, as an individual H3K27 is either trimethylated or not. At the nucleosome level, there can be no, one, or two H3K27 tails with a trimethyl modification. At the cell population level, there can be considerable quantitative differences because of the heterogeneity for histone modification status between individual cells. Cell variability may be the source of the quantitative differences observed in our experiments, arising from variation in both the percentage of cells with H3K27me3 and the number of tails of a nucleosome with H3K27me3. While we cannot determine this based on our present data, our results show that some genes that are differentially expressed between growth conditions are associated with quantitative differences in H3K27me3 levels, providing evidence that chemical histone modification dynamics can be involved in a transcriptional regulatory mechanism in V. dahliae. The concept of local versus global H3K27me3 changes is consistent with data from M. oryzae, in which direct *in planta* H3K27me3 ChIP-quantitative PCR (qPCR) showed that some, but not all, analyzed regions displayed differential H3K27me3 levels consistent with increased transcription between *in planta* and *in vitro* conditions (50). We have not been able to directly assess histone modifications for V. dahliae during plant infection, but we did analyze the status of H3K27 methylation during *in vitro* growth of *in planta*-induced genes. The *in planta*-induced genes have significantly higher levels of H3K27me3 and the DNA is substantially less accessible during *in vitro* growth than genes that are highly expressed during *in vitro* growth. We conclude that the dynamics for H3K27me3 can contribute to differential gene expression under natural conditions, but our results show that this is not required. Our conclusions are limited to the tested conditions, and it is possible that analyzing H3K27me3 levels in different cell types or growth stages (e.g., spores, microsclerotia, and *in planta* infection) may yield a different picture of the global H3K27me3 distribution. Additionally, our experiments focused on steady-state growth on different media as these provide a clear and reproducible transcriptional difference. It is possible that H3K27me3 dynamics occur rapidly in response to environmental changes, cues, or developmental stages that we did not capture.

It appears evident that H3K27me3 contributes to additional genome functions beyond strict transcriptional repression, at least in fungi (37). This is supported by our data showing that the majority of H3K27me3 domains were stable between the tested growth conditions, indicating that these H3K27me3 domains have additional functions. Stable H3K27me3 domains may represent another form of constitutive heterochromatin, but this seems unlikely given that in our data, many stable H3K27me3 domains harbored differentially expressed genes between growth media. Additionally, results for N. crassa have shown that genetic perturbation leading to the genome-wide loss of H3K9me3 causes a redistribution of H3K27me3 to previously H3K9me3-marked sites, but interestingly, this redistribution does not result in transcriptional silencing (53). Rather, the redistribution of H3K27me3 in N. crassa lacking H3K9me3 appears to contribute to genomic instability (53, 54). In *Z. tritici*, the enrichment of H3K27me3 at dispensable chromosomes and empirical evidence that H3K27me3 somehow increases genomic instability additionally support the hypothesis that H3K27me3 contributes to additional genomic functions beyond transcriptional regulation (32, 48). Evolutionary analysis across Fusarium and related species indicates that genes marked by H3K27me3 have a higher duplication rate in Fusarium and are less conserved with more distantly related species (55). Additional research is needed to fully understand the mechanisms of H3K27me3 targeting, dynamics, and impact on genome stability in fungi. The results presented here show that H3K27me3 domains are largely similar between *in vitro* growth conditions but that quantitative differences in H3K27me3 levels can be associated with concomitant transcriptional differences between the tested conditions.

## MATERIALS AND METHODS

### Fungal growth conditions.

Verticillium dahliae strain JR2 (CBS 143773) (56) was cultured on potato dextrose agar (PDA) (Oxoid, Thermo Scientific) at 22°C in the dark. Liquid cultures were obtained by collecting conidiospores from PDA plates after approximately 2 weeks of growth, followed by inoculation at a final concentration of 1 × 10^4^ spores per mL into liquid growth media. Media used in this study are potato dextrose broth (PDB) (Difco, Becton, Dickinson, Franklin Lakes, NJ, USA), half-strength Murashige-Skoog medium plus vitamins (MS) (Duchefa-Biochemie, Haarlem, The Netherlands) supplemented with 3% sucrose, and Czapek-Dox medium (CZA) (Oxoid, Thermo Scientific, Waltham, MA, USA). Liquid cultures were grown for 6 days in the dark at 22°C at 140 rpm. The mycelium was collected by straining cultures through Miracloth (22 μm) (EMD Millipore, Darmstadt, Germany) and pressing to remove liquid, after which the mycelium was flash-frozen in liquid nitrogen and ground to a powder with a mortar and pestle. If required, samples were stored at −20°C prior to nucleic acid extraction. All analyses were performed based on triplicate cultures that were processed individually.

### RNA sequencing and analysis.

RNA was isolated from ground mycelium using TRIzol (Thermo Fisher Scientific, Waltham, MA, USA) according to the manufacturer’s guidelines. Contaminating DNA was removed using the Turbo DNA-free kit (Ambion, Thermo Fisher Scientific, Waltham, MA, USA), and RNA integrity was assessed by gel electrophoresis and quantified using a Nanodrop spectrophotometer (Thermo Fisher Scientific, Waltham, MA, USA). Libraries were prepared and single-end 50-bp sequenced on the DNBseq platform at BGI (Hong Kong, China).

Sequencing reads were mapped to the reference annotation of V. dahliae strain JR2 (56) using Kallisto quant (57) to calculate per-gene transcripts per million (TPM) values. Differential expression between cultivation in PDB and CZA or MS or during colonization of Arabidopsis thaliana was determined using DESeq2 (58).

### ChIP sequencing and analysis.

ChIP-seq was performed as described previously (44). The frozen ground mycelium was added to ChIP lysis buffer, homogenized in a Dounce homogenizer 40 times, and subsequently sonicated for 5 rounds of 20 s with 40-s rest stages on ice. Supernatants were collected after centrifugation and treated with micrococcal nuclease (MNase) (New England BioLabs, Ipswich, MA, USA) for 10 min in a 37°C water bath. MNase activity was quenched by the addition of EGTA, and samples were subsequently precleared by the addition of 40 μL protein A magnetic beads (New England BioLabs, Ipswich, MA, USA) and rotation at 4°C for 60 min. Beads were captured, and the supernatant was divided over new tubes containing antibodies against either H3K4me2, H3K9me3, or H3K27me3 (catalog numbers 39913, 39765, and 39155; ActiveMotif) and incubated overnight with continuous rotation at 4°C. Subsequently, the antibodies were captured and washed, and nucleosomes were eluted from the beads, after which DNA was treated with proteinase K and cleaned up using chloroform. DNA was isolated by overnight precipitation in ethanol, and the DNA concentration was determined with the Qubit dsDNA HS (double-stranded DNA high-sensitivity) assay kit (Thermo Fisher Scientific, Waltham, MA, USA). Sequencing libraries were generated using the TruSeq ChIP library preparation kit (Illumina, San Diego, CA, USA) according to the manufacturer’s instructions but without gel purification and with the use of the Velocity DNA polymerase (BioLine, Luckenwalde, Germany) for 25 cycles of amplification. Single-end 125-bp sequencing was performed on the Illumina HiSeq2500 platform at KeyGene NV (Wageningen, The Netherlands).

Raw reads were trimmed using TrimGalore (59) and mapped to the V. dahliae strain JR2 reference genome (56) using BWA-mem with default settings (60). Three regions of the genome were masked due to aberrant mapping, (chromosome 1 positions 1 to 45000 [chr1:1–45000], chr2:3466000–3475000, and chr3:1–4200). ATAC-seq reads of V. dahliae cultured in PDB (44) were treated similarly to the ChIP-seq reads, but the paired-end read pairs were trimmed and mapped simultaneously, and only read pairs of <100 bp were considered for further analyses, as these represent open DNA. Mapped reads were normalized to reads per genomic content (RPGC) using deepTools bamCoverage (61), with the settings binsize 1,000 and smoothlength 3,000 for plotting over the genome and the settings binsize 10 and smoothlength 30 for further analysis. Normalized replicate samples with high correlation were selected, and mean data sets per growth medium were generated with input controls for background signal correction using WiggleTools mean (62). H3K27me3-enriched regions were determined on selected replicates with input control using epic2 with a bin size of 2,500 bp (63). The average coverage over gene bodies per expression quintile was calculated using deepTools computeMatrix in scale-regions mode (61). Expression quintiles were generated by sorting all genes based on their average TPM values from three replicates of V. dahliae cultured for 6 days in PDB. We used deepTools multiBigwigSummary (61) to determine the presence of H3K27me3 over gene bodies (region between the TSS and TES) for each replicate ChIP sample as well as for input controls. Samples and input controls were TPM normalized, after which the normalized input control signal was subtracted from the normalized H3K27me3 TPM values, and the resulting negative values were set to zero. Changes in H3K27me3 levels between growth media were determined by taking the average input-normalized H3K27me3 TPM values plus 1 and calculating the log_2_ fold change for each pairwise comparison.

### Generation of the *Set7* deletion mutant.

The *Set7* deletion mutant (Δ*Set7*) was constructed as previously described (64). Briefly, genomic DNA regions flanking the 5′ and 3′ ends of the coding sequences were amplified by PCR using primers listed in Table S1 in the supplemental material (primers 1 to 4) and cloned into the pRF-HU2 vector (65) using User enzyme according to the manufacturer’s protocol (New England BioLabs, MA, USA). Sequence-verified vectors were transformed into Agrobacterium tumefaciens strain AGL1 and used for V. dahliae conidiospore transformation as described previously (64). V. dahliae transformants that appeared on hygromycin B were transferred to fresh PDA supplemented with hygromycin B after 5 days. Putative transformants were screened using PCR to verify the deletion of the target gene sequence and the integration of the selection marker at the designated locus using primers listed in Table S1 (primers 5 to 8). Analysis and comparison of gene expression in the *Set7* deletion mutant to that in wild-type V. dahliae were performed as described above.

10.1128/mbio.03566-21.9 TABLE S1 Primers used to delete and analyze the *Set7* coding sequence in V. dahliae. Download Table S1, PDF file, 0.06 MB. Copyright © 2022 Kramer et al. 2022 Kramer et al. https://creativecommons.org/licenses/by/4.0/ This content is distributed under the terms of the Creative Commons Attribution 4.0 International license.
