# Supplementary material for: Local Rather than Global H3K27me3 Dynamics Are Associated with Differential Gene Expression in Verticillium dahliae
Source: mBio. 2022 Feb 8;13(1):e03566-21. doi: 10.1128/mbio.03566-21 (PMC8822345; doi:10.1128/mbio.03566-21)
Supplement: FIG S1 [file mbio.03566-21-sf001.pdf]

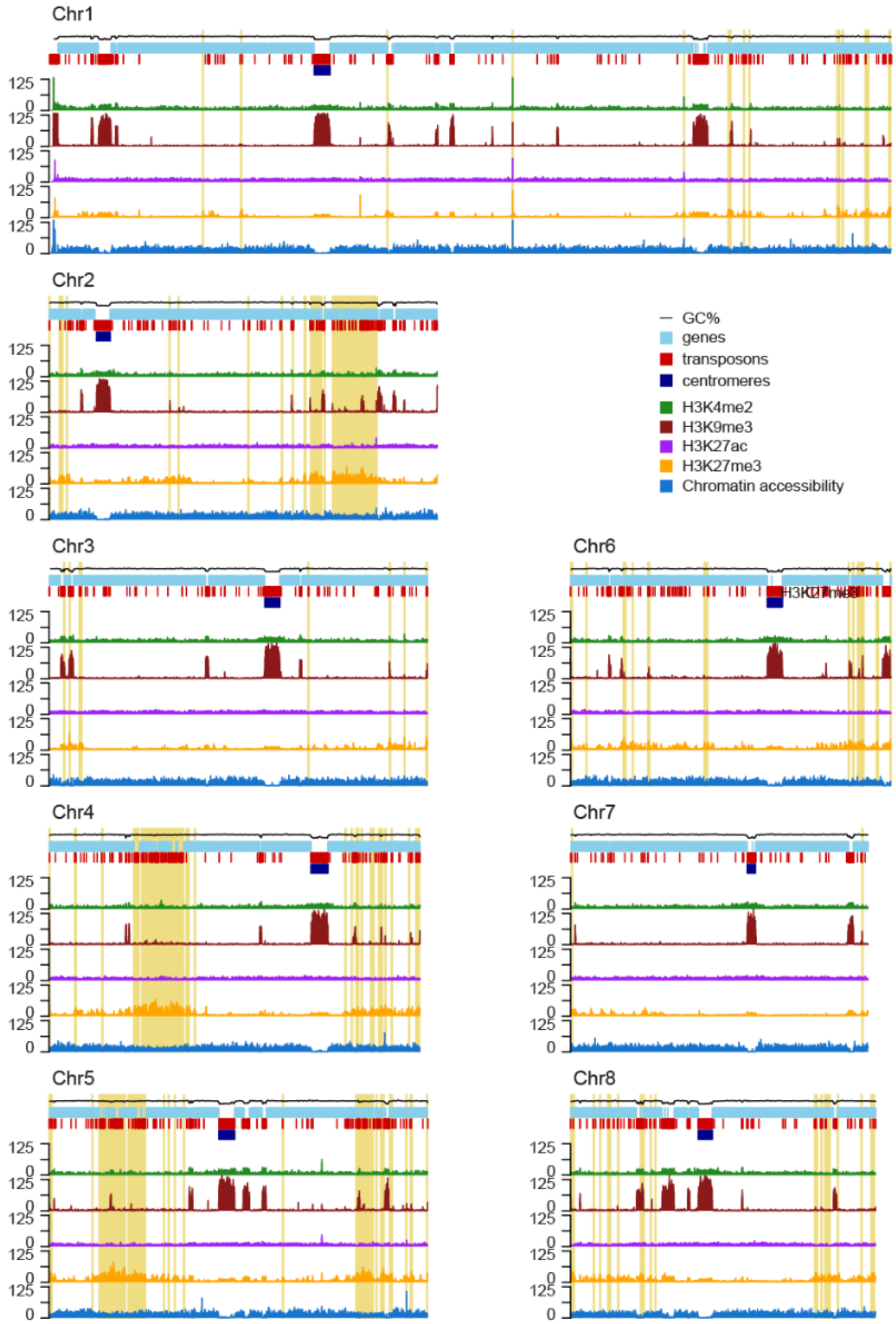

Figure S1. Distribution of chromatin features over all chromosomes. Whole genome distribution of the euchromatin-associated histone modification H3K4me2 (green line), the constitutive heterochromatin-associated histone modification H3K9me3 (red line), the facultative heterochromatin-associated histone modification H3K27me3 (yellow line) and chromatin accessibility (blue line) as determined by ATAC-seq. Genes are indicated in light blue, transposons are indicated in red, centromere is indicated in dark blue and adaptive genomic regions are indicated in yellow.
