# Supplementary material for: Local Rather than Global H3K27me3 Dynamics Are Associated with Differential Gene Expression in Verticillium dahliae
Source: mBio. 2022 Feb 8;13(1):e03566-21. doi: 10.1128/mbio.03566-21 (PMC8822345; doi:10.1128/mbio.03566-21)
Supplement: FIG S2 [file mbio.03566-21-sf002.pdf]

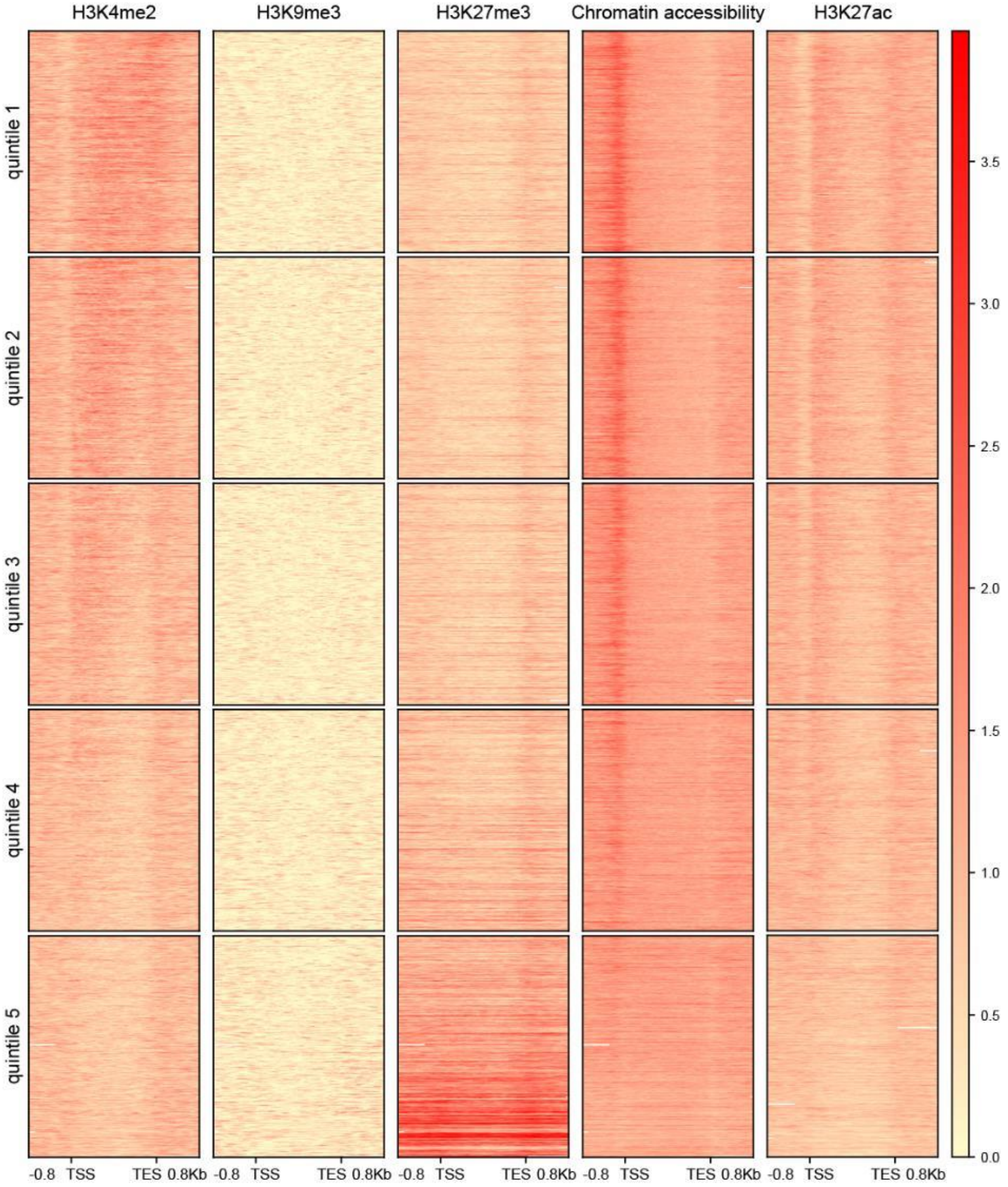

Figure S2. Gene expression correlates with histone modification presence and chromatin accessibility. RPGC normalized coverage of the histone marks H3K4me2, H3K9me3, H3K27me3 and chromatin accessibility over gene bodies (between transcription start site (TSS) and transcription end site (TES)) ±800 bp of flanking sequence. Each row represents a single gene, and are sorted based on their TPM value upon cultivation for six days potato dextrose broth (PDB), with the top gene being most highly expressed. Genes are grouped in expression quintiles as in Figure 3A.
