# Supplementary material for: Local Rather than Global H3K27me3 Dynamics Are Associated with Differential Gene Expression in Verticillium dahliae
Source: mBio. 2022 Feb 8;13(1):e03566-21. doi: 10.1128/mbio.03566-21 (PMC8822345; doi:10.1128/mbio.03566-21)
Supplement: FIG S3 [file mbio.03566-21-sf003.pdf]

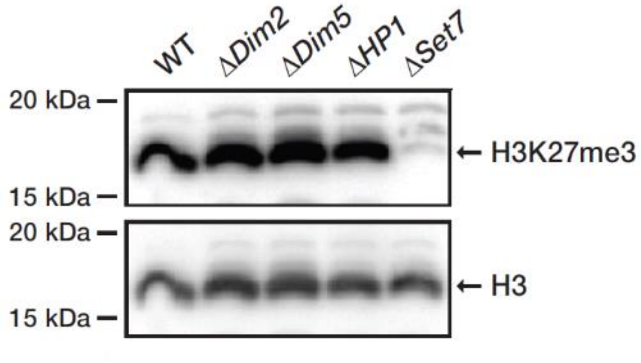

Figure S3. Western blot shows loss of H3K27me3 in the V. dahliae Set7 deletion mutant. Histone isolations of wild-type, ΔDim2, ΔDim5, ΔHP1 and ΔSet7 were tested for presence of the H3K27me3 histone modification by Western blotting. The antibody against H3 was used as loading control.
