# Supplementary material for: Local Rather than Global H3K27me3 Dynamics Are Associated with Differential Gene Expression in Verticillium dahliae
Source: mBio. 2022 Feb 8;13(1):e03566-21. doi: 10.1128/mbio.03566-21 (PMC8822345; doi:10.1128/mbio.03566-21)
Supplement: FIG S4 [file mbio.03566-21-sf004.pdf]

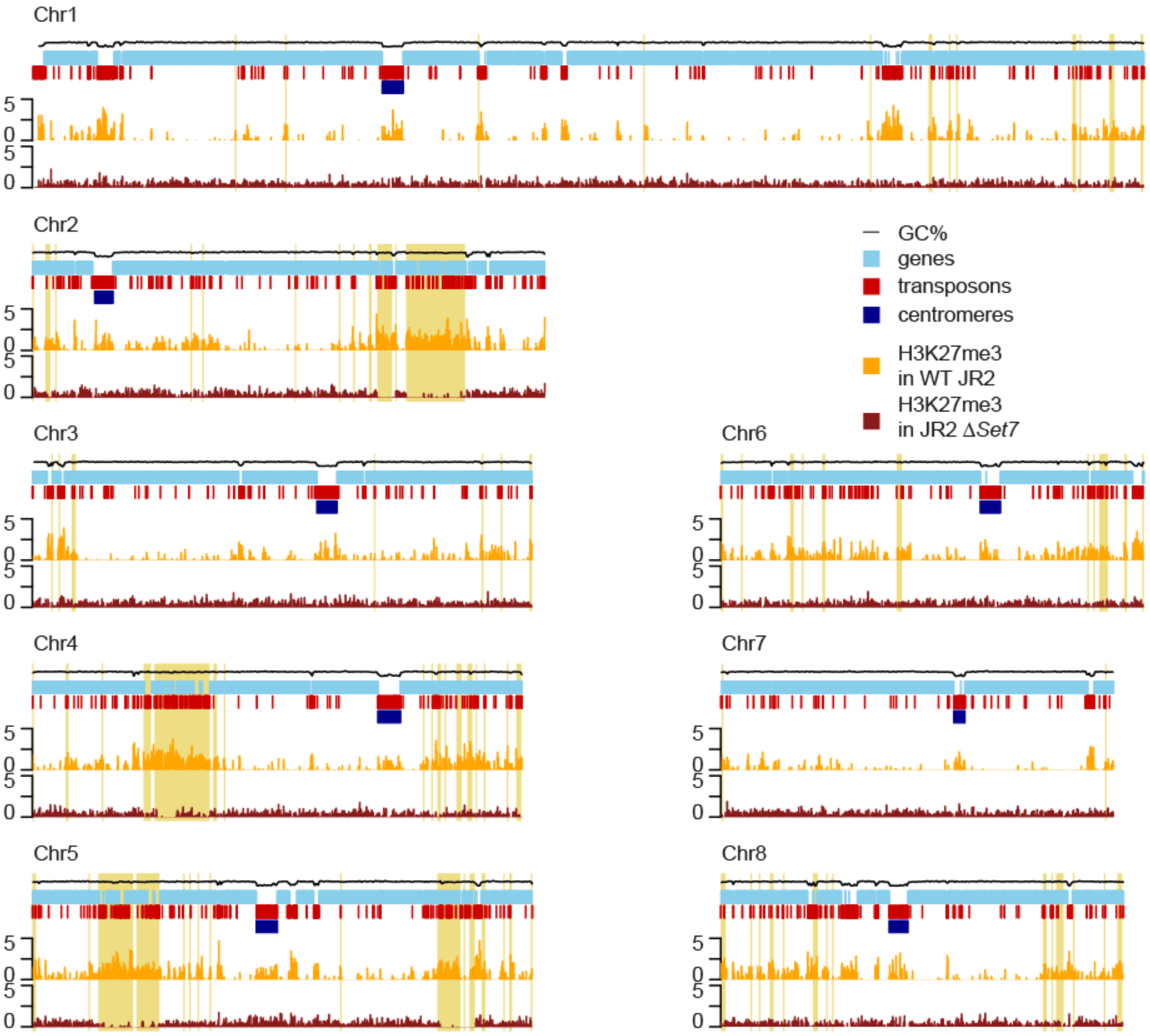

Figure S4. ChIP-sequencing shows loss of H3K27me3 in the V. dahliae ΔSet7 mutant. H3K27me3 ChIP coverage over the genome in a triplicate of JR2 WT (yellow) and in a duplicate of JR2 ΔSet7, cultivated for 6 days in potato dextrose broth.
