# Supplementary material for: Local Rather than Global H3K27me3 Dynamics Are Associated with Differential Gene Expression in Verticillium dahliae
Source: mBio. 2022 Feb 8;13(1):e03566-21. doi: 10.1128/mbio.03566-21 (PMC8822345; doi:10.1128/mbio.03566-21)
Supplement: FIG S5 [file mbio.03566-21-sf005.pdf]

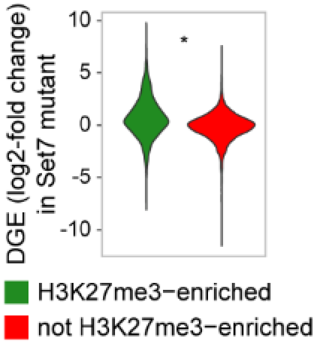

Figure S5. Genes associated with H3K27me3 in wild type V. dahliae are stronger transcriptionally induced in the ΔSet7 mutant than not H3K27me3 associated genes. Log2-fold change of expression between wild type and ΔSet7 mutant for genes associated with H3K27me3 in wild type (green) and those not associated with H3K27me3 in wild type (red). Significant difference of log2-fold change between gene sets are determined with the One-Sample Wilcoxon Signed Rank Test (*: p <= 0.05).
