# Supplementary material for: Local Rather than Global H3K27me3 Dynamics Are Associated with Differential Gene Expression in Verticillium dahliae
Source: mBio. 2022 Feb 8;13(1):e03566-21. doi: 10.1128/mbio.03566-21 (PMC8822345; doi:10.1128/mbio.03566-21)
Supplement: FIG S6 [file mbio.03566-21-sf006.pdf]

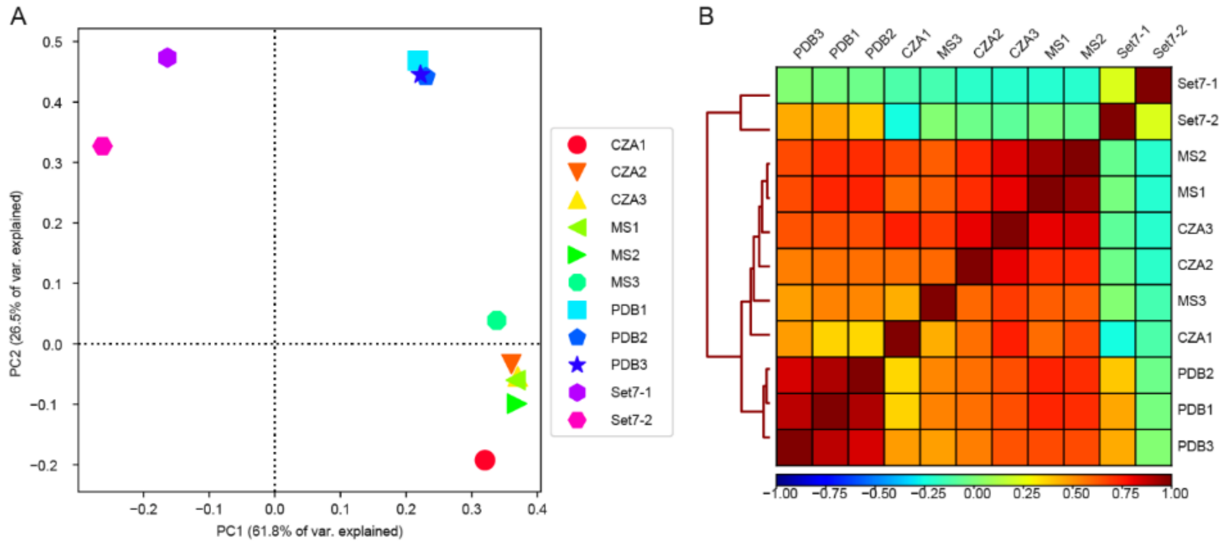

Figure S6. Correlation between H3K27me3 ChIP samples. A) PCA plot and B) heatmap displaying between-sample correlation of H3K27me3 coverage for 1kb bins for triplicates of JR2 WT cultivated for 6 days in Czapec-Dox medium (CZA), half-strength Murashige-Skoog medium (MS) and potato dextrose broth (PDB), and a duplicate JR2 ΔSet7 cultivated for 6 days in PDB.
