# Supplementary material for: Local Rather than Global H3K27me3 Dynamics Are Associated with Differential Gene Expression in Verticillium dahliae
Source: mBio. 2022 Feb 8;13(1):e03566-21. doi: 10.1128/mbio.03566-21 (PMC8822345; doi:10.1128/mbio.03566-21)
Supplement: FIG S7 [file mbio.03566-21-sf007.pdf]

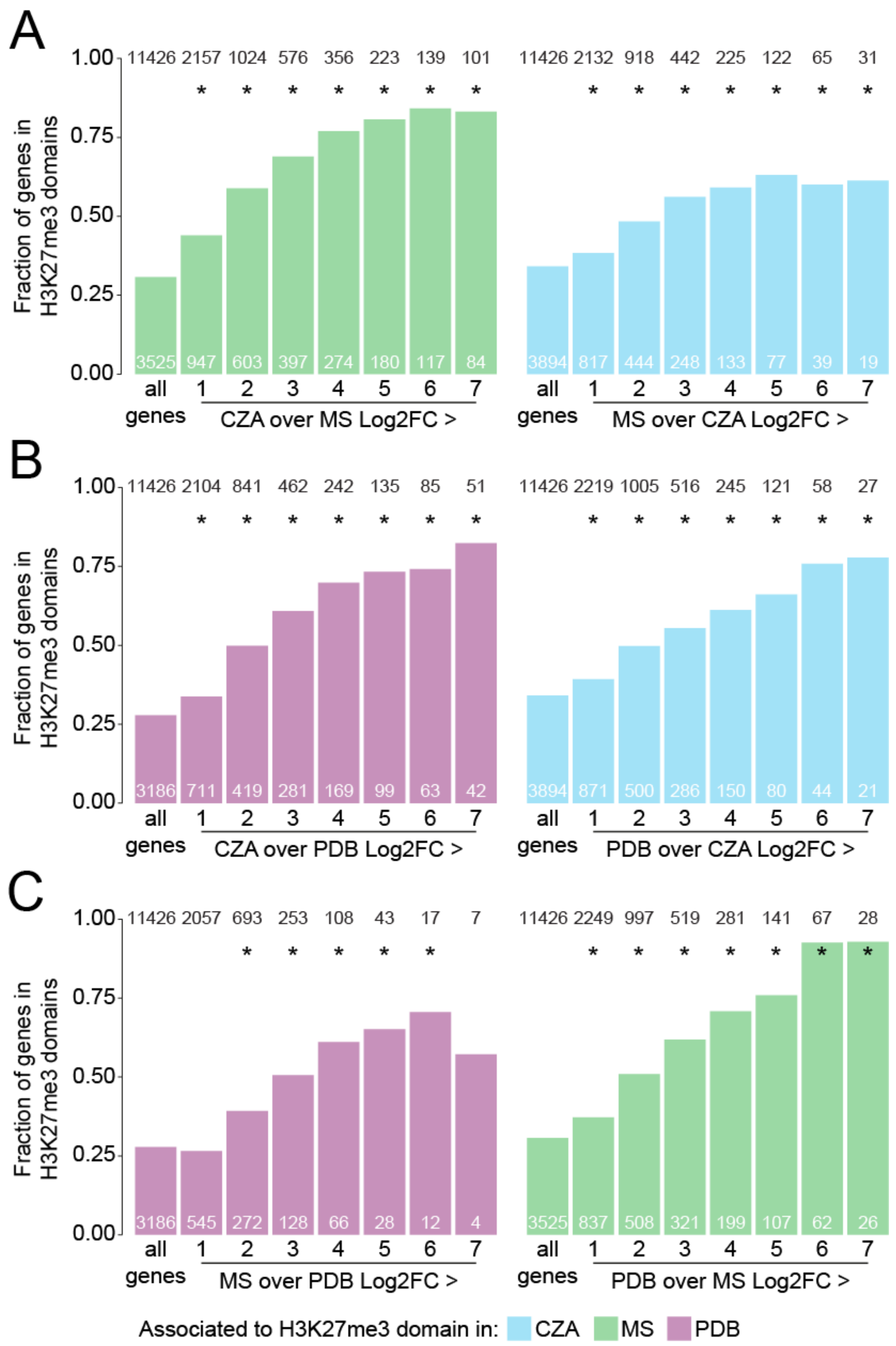

Figure S7. Differentially expressed genes are enriched in H3K27me3 domains during cultivation in the non-permissive growth medium. Fractions of total and differentially expressed genes (Log2-fold changes >1, 2, 3, 4, 5, 6 and 7) that are associated with an H3K27me3 domain in the non-permissive condition Czapec-Dox medium (CZA, blue bars), half-strength Murashige-Skoog medium (MS, green bars) or Potato-Dextrose broth (PDB, red bars) between (A) cultivation for 6 days in CZA and MS, (B) cultivation for 6 days in CZA and PDB and (C) cultivation for 6 days in MS and PDB. Black numbers above the bars indicate numbers of genes per gene set. White numbers at bottom of the bars indicate the numbers of genes that locate in an H3K27me3-domain per gene set. Significance of fraction differences was calculated for each fraction of

higher expressed genes in adaptive genomic regions when compared with the fraction of total genes in adaptive genomic regions, by the two-sided two-proportions Z-test (*: $p \leq 0.05$).
