# Supplementary material for: Local Rather than Global H3K27me3 Dynamics Are Associated with Differential Gene Expression in Verticillium dahliae
Source: mBio. 2022 Feb 8;13(1):e03566-21. doi: 10.1128/mbio.03566-21 (PMC8822345; doi:10.1128/mbio.03566-21)
Supplement: FIG S8 [file mbio.03566-21-sf008.pdf]

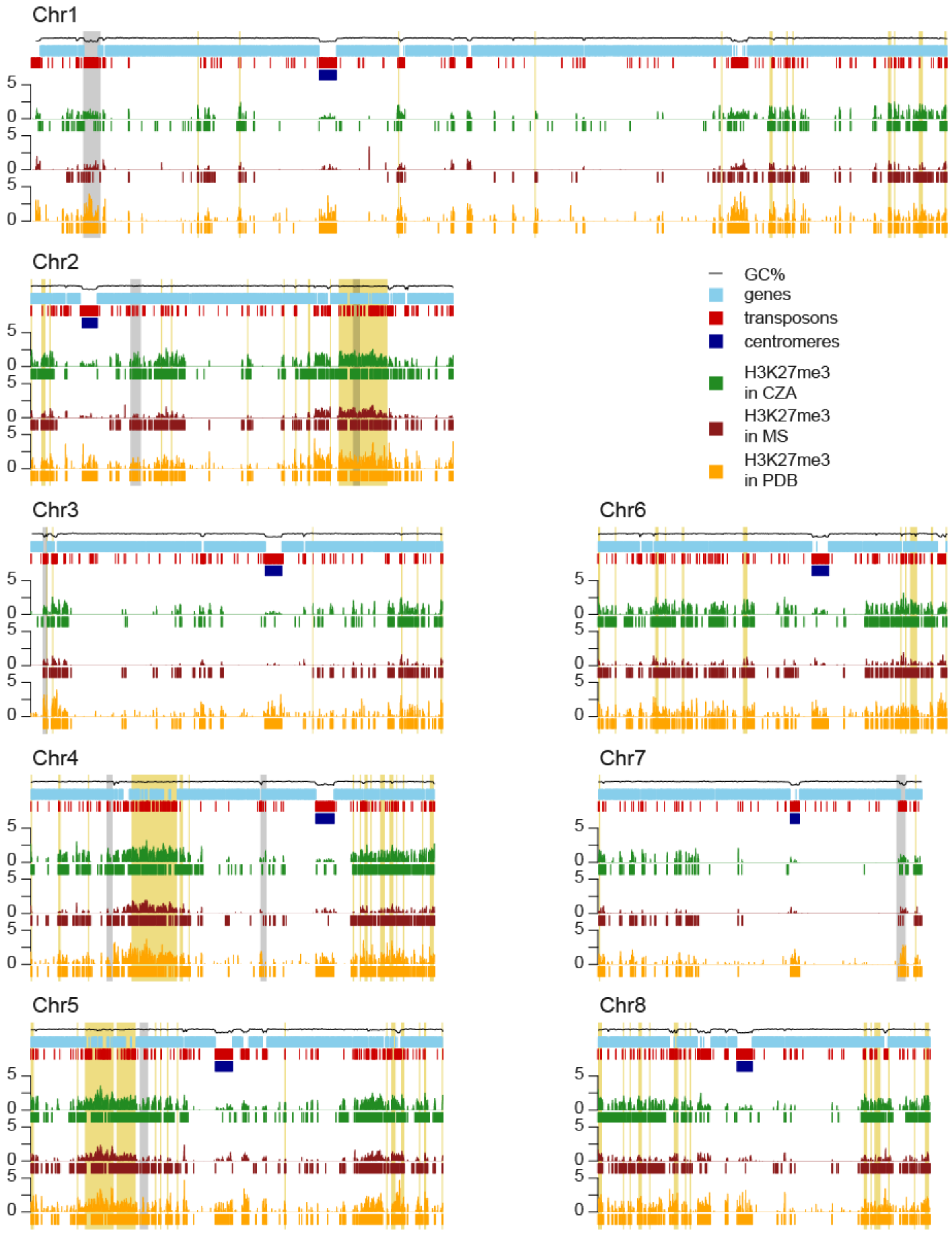


***Figure S8. Distribution of H3K27me3 for*** **V. dahliae** ***cultivated*** **in vitro*.*** *Average H3K27me3 distribution for replicates of* V. dahliae *cultivated for 6 days in potato dextrose broth (PDB, indicated in yellow), half strength Murashige Skoog medium (MS, indicated in red) and Czapec-Dox medium (CZA, indicated in green). Predicted H3K27me3 domains are indicated as blocks below each H3K27me3 track. Adaptive genomic regions are highlighted in yellow. Genomic regions within H3K27me3 domains that are visibly different between growth conditions are highlighted in grey.*
