# Supplementary material for: Local Rather than Global H3K27me3 Dynamics Are Associated with Differential Gene Expression in Verticillium dahliae
Source: mBio. 2022 Feb 8;13(1):e03566-21. doi: 10.1128/mbio.03566-21 (PMC8822345; doi:10.1128/mbio.03566-21)
Supplement: TABLE S1 [file mbio.03566-21-st001.pdf]

Table S1: Primers used to delete and analyze the Set7 coding sequence in *V. dahliae*.

Name	Sequence	Purpose	Number
Set7.ko-LB_F	*GGTCTTAAU*TGAGCTTGACAGTTCAGTTGTCG	Amplify left flanking sequence	1
Set7.ko-LB_R	*GGCATTAAU*AAGTTGTGTTGTCAGCGTGCATA	Amplify left flanking sequence	2
Set7.ko-Rb_F	*GGACTTAAU*ATCAAGTCCGCCTACTTTCCAAG	Amplify right flanking sequence	3
Set7.ko-Rb_F	*GGGTTTAAU*GTGGAGAATCGTCTGGGGTTATC	Amplify right flanking sequence	4
Set7_Confirm_F	CCTCCAGCTCCTGAAGAAGAA	Confirm gene replacement (selection)	5
Vector_Reverse	GGAGTCGCATAAGGGAGAGCG	Confirm gene replacement (selection)	6
Set7.ORF.270_F	TCACAGCCGCTACATCAATCA	Confirm ORF is absent in KO	7
Set7.ORF.270_R	TCGTGTTGAACCTCCTTGGAC	Confirm ORF is absent in KO	8

Italic sequences at the 5' ends represent adapters added for USER cloning
